# Structural basis for activation of plasma-membrane Ca^2+^-ATPase by calmodulin

**DOI:** 10.1038/s42003-018-0203-7

**Published:** 2018-11-26

**Authors:** Julius Nitsche, Inokentijs Josts, Johannes Heidemann, Haydyn D. Mertens, Selma Maric, Martine Moulin, Michael Haertlein, Sebastian Busch, V. Trevor Forsyth, Dmitri I. Svergun, Charlotte Uetrecht, Henning Tidow

**Affiliations:** 1 The Hamburg Centre for Ultrafast Imaging & Department of Chemistry, Institute for Biochemistry and Molecular Biology, University of Hamburg, Martin-Luther-King-Platz 6, 20146 Hamburg, Germany; 2 Heinrich Pette Institute, Leibniz Institute for Experimental Virology, Martinistrasse 52, 20251 Hamburg, Germany; 3 European Molecular Biology Laboratory Hamburg, Notkestrasse 85, 22607 Hamburg, Germany; 4 Biofilms- Research Center for Biointerfaces, Department of Biomedical Science, Faculty of Health and Society, Malmö University, Malmö, 20506 Sweden; 5 Life Sciences Group, Institut Laue–Langevin, 6 Rue Jules Horowitz, 38042 Grenoble, France; 6 German Engineering Materials Science Centre (GEMS) at Heinz Maier-Leibnitz Zentrum (MLZ), Helmholtz-Zentrum Geesthacht, Lichtenbergstr. 1, 85747 Garching bei München, Germany; 7 School of Life Sciences, Keele University, Staffordshire, ST5 5BG England; 8 European XFEL GmbH, Holzkoppel 4, 22869 Schenefeld, Germany

**Keywords:** SAXS, Membrane proteins

## Abstract

Plasma-membrane Ca^2+^-ATPases expel Ca^2+^ from the cytoplasm and are key regulators of Ca^2+^ homeostasis in eukaryotes. They are autoinhibited under low Ca^2+^ concentrations. Calmodulin (CaM)-binding to a unique regulatory domain releases the autoinhibition and activates the pump. However, the structural basis for this activation, including the overall structure of this calcium pump and its complex with calmodulin, is unknown. We previously determined the high-resolution structure of calmodulin in complex with the regulatory domain of the plasma-membrane Ca^2+^-ATPase ACA8 and revealed a bimodular mechanism of calcium control in eukaryotes. Here we show that activation of ACA8 by CaM involves large conformational changes. Combining advanced modeling of neutron scattering data acquired from stealth nanodiscs and native mass spectrometry with detailed dissection of binding constants, we present a structural model for the full-length ACA8 Ca^2+^ pump in its calmodulin-activated state illustrating a displacement of the regulatory domain from the core enzyme.

## Abstract (web summary)

Julius Nitsche et al. demonstrate that binding of two calmodulin molecules displaces the regulatory domain of the plasma-membrane Ca^2+^-ATPase ACA8 to fully activate this Ca^2+^ pump by relieving the autoinhibition. This work provides structural evidence for the previously proposed bimodular activation mechanism.

## Introduction

Calcium ions (Ca^2+^) play a major role as secondary messengers in numerous signal transduction processes (reviewed in the ref. ^1,2^). In order to maintain the concentration gradient between low intracellular (100 nM) and high extracellular (2 mM) Ca^2+^ that sets the stage for calcium signaling^1^, eukaryotic cells have evolved a sophisticated regulation system involving plasma-membrane Ca^2+^-ATPases (PMCAs). These high-affinity Ca^2+^ pumps export Ca^2+^ ions from the cytosol into the extracellular environment using energy provided through ATP hydrolysis, and are tightly regulated. They help to maintain overall Ca^2+^ homeostasis and provide local control of intracellular Ca^2+^ signaling^3,4^.

PMCAs belong to the P_2_B subfamily of P-type ATPases^5^ and play a major physiological role in e.g., for pre-synaptic and post-synaptic Ca^2+^ regulation in neurons, feedback signaling in the heart and sperm motility^6^. Compared to other P-type ATPases, plasma-membrane calcium ATPases contain an additional autoinhibitory or regulatory domain. While mammalian PMCAs contain a C-terminal autoinhibitory domain, in plant ACAs (autoinhibited Ca^2+^-ATPases) this domain is located at the N-terminus^7^. Binding of calmodulin (Ca^2+^-CaM) to this region relieves autoinhibition and results in pump activation, however, the conformational changes leading to PMCA activation are not well understood.

To investigate the structural basis for PMCA activation, we have dissected the underlying binding events of autoinhibition and activation using biochemical and biophysical methods, and combined this analysis with small-angle neutron and X-ray scattering studies of a PMCA pump reconstituted in stealth nanodiscs, facilitating its structural characterization in a lipidic solution environment. Using this hybrid approach, we developed a structural model for the activation of plasma-membrane Ca^2+^-ATPases by calmodulin. In its calmodulin-activated state, the regulatory domain is displaced from the core, enabling maximal enzymatic activity. The combination of stealth nanodiscs, selectively deuterated protein components and SANS should be broadly applicable to many membrane protein complexes.

## Results

### Binding of two calmodulin molecules to ACA8 leads to its activation

**Fig. 1:**
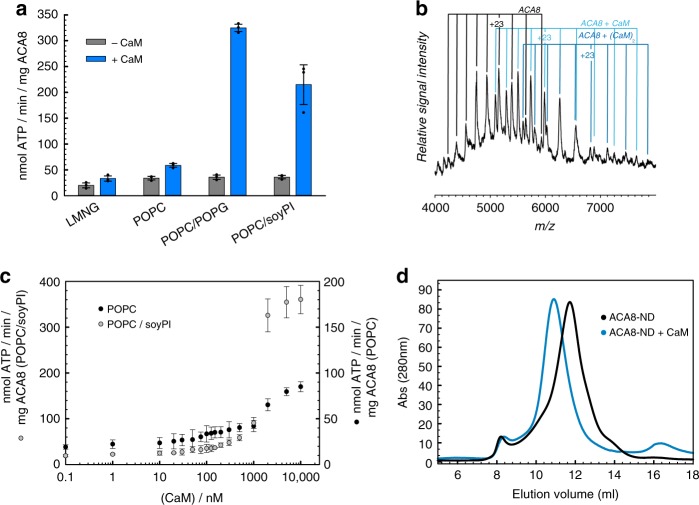
Incorporation of ACA8 in nanodiscs and activation by CaM-binding. **a** ACA8 ATPase activity assay. Activity was measured (in triplicates) in detergent micelles and nanodiscs containing different lipids in absence or presence of calmodulin. **b** Native mass spectrometry showing up to two CaM molecules bound to full-length ACA8 in detergent solubilized state. At high acceleration voltages (200 V) proteins are released from the detergent micelles. The masses (Supplementary Table 1) indicate the presence of unbound ACA8 (black), ACA8-CaM (light blue) and ACA8-(CaM)_2_ (dark blue). **c** CaM-dependent activity measurements of nanodisc-incorporated ACA8. Measurements were performed in nanodiscs containing POPC (black) or POPC/soyPI mixture (gray) as lipid component with different amounts of CaM added. **d** Size-exclusion chromatography profile of ACA8 in nanodisc (black line) and in complex with CaM (blue line) showing an expansion once CaM is bound

Full-length ACA8 protein was expressed and purified to homogeneity and its state of inhibition was assessed by the basal ATPase activity. Binding of calmodulin (CaM7, hereafter denoted as CaM) to the CaM-binding domain (CaMBD) of detergent solubilized ACA8 leads to a two-fold increase in ATPase activity of the pump compared to the basal activity in the absence of CaM (Fig. 1a). To gain insights into the stoichiometry of the ACA8-CaM complexes, DDM-solubilized and purified ACA8 in its apo and CaM-bound states was analyzed by native mass spectrometry. Native mass spectrometry is an established method for the precise determination of protein stoichiometries in soluble and membrane protein complexes^8^. The spectrum shows peaks corresponding to the apo ACA8, ACA8-CaM (single CaM) and ACA8-CaM_2_ (two CaM molecules bound) (Fig. 1b). In the full *m/z* range spectrum highly charged CaM is detected, which dissociated from the weakly charged ACA8 and the ACA8-CaM complex (Supplementary Fig. 1). These findings show that a maximum of two CaM molecules can bind to the full-length ACA8 as previously observed for the isolated regulatory domain and thereby confirm the proposed two-step activation mechanism^9^.

In order to investigate the structure and conformational changes of ACA8 in a native-like lipidic environment, we reconstituted the pump in MSP1D1 nanodiscs (ND) composed of various lipids (Supplementary Fig. 2). Size-exclusion chromatography profiles of ACA8 incorporated in nanodiscs for all different types of lipids indicated a monodisperse sample preparation (Fig. 1d). ATP hydrolysis assays in different lipid reconstitutions revealed much higher activity of CaM-activated ACA8 in the nanodiscs composed of 1-palmitoyl-2-oleoyl-sn-glycero-3-phosphocholine (POPC) compared to the activity of the ACA8-CaM complex in detergent micelles (Fig. 1a), emphasizing the importance of a lipidic environment for the proper function and activation of ACA8 as previously demonstrated for human PMCAs^10,11^. Reconstitution of ACA8 into nanodiscs containing 70% POPC and 30% POPG or 70% POPC and 30% soy-PI results in a further 3–5-fold activity increase of CaM-activated ACA8 compared with POPC alone, showing that negatively charged head groups are able to further increase the enzymatic activity once the pump is activated (Fig. 1a). Overall, our absolute activity values as well as the activation factors (activity of ACA8 + CaM/activity of ACA8) for nanodisc-incorporated samples are in good agreement with previous results obtained using microsome preparations^12–15^. To further improve our understanding of the role of lipids in ACA8 activation by CaM we analyzed the basal ATPase activity of ACA8 by titrating CaM to the apo-ACA8 in POPC and POPC/soy-PI nanodiscs. In both instances, we observe concentration-dependent increase in the ATP hydrolysis rate, revealing two transitions, which reflect the proposed bimodular activation mechanism^9^ (Fig. 1c). Additionally, these experiments show a marked effect of ACA8 activation by negatively charged lipids at higher CaM concentrations. Above a CaM concentration of approx. 500 nM, the activity of ACA8 in nanodiscs composed of soy-PI is shifted to higher pump rates, indicating that once two CaM molecules are bound, the anionic headgroup of soy-PI further stimulates the activity. These findings are in good agreement with previous studies on PMCAs with a photo-activatable phosphatidylcholine analog, showing that CaM and phosphatidic acid behave independently regarding their effects on enzyme activity and transmembrane conformation^16^. Altogether, these results support the bimodular activation mechanism and further show the importance of diverse surrounding lipids for the activation process.

### CaM-binding induces large conformational changes in ACA8

**Fig. 2:**
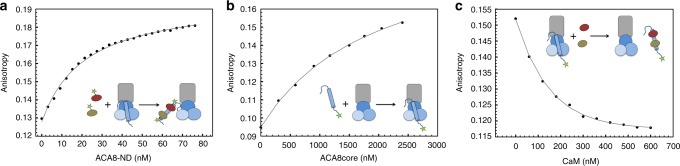
Binding affinities within ACA8-CaM complex investigated by fluorescence anisotropy. **a** Binding of fluorescein-labeled CaM to full-length ACA8 (*K*_d_ = 18 nM). **b** Binding of fluorescein-labeled regulatory domain (RD) to ACA8core (*K*_d_ = 1.7 μM). **c** Addition of CaM to a preformed complex containing ACA8core and labeled RD. Decrease in anisotropy indicates displacement of RD from ACA8core through CaM-binding to RD. All experiments were performed using protein reconstituted in nanodiscs

In order to investigate the structural changes leading to the activation of ACA8 by CaM, we carefully dissected the different binding affinities of the various protein-protein interactions during activation by using fluorescence anisotropy (Fig. 2). Fluorescence anisotropy detects differences in rotational speed, which in turn correlates with differences in molecular masses (if the mass differences are large enough). All experiments were performed using ACA8 constructs reconstituted in POPC-nanodiscs.

We first measured binding of fluorescein-labeled CaM to full-length ACA8, revealing tight binding with an apparent *K*_d_ of 18 nM (Fig. 2a). In this setup, the measured affinity corresponds to binding of CaM to the high-affinity binding site and resembles the value previously determined for CaM-binding to the isolated regulatory domain^9^. To characterize the structural changes during the activation we expressed and purified the regulatory domain of ACA8 (ACA8RD, residues 1–130) alone in *E. coli*. Next, we assessed the level of secondary structure in ACA8RD using far-UV circular dichroism spectroscopy. These experiments indicated that this domain consists partly of random coil segments with some degree of α-helicity, presumably spanning residues 40–95 as observed in the ACA8RD-(CaM)_2_ crystal structure^9^ (pdb:4aqr) (Supplementary Fig. 3). Titration of the fluorescein-labeled ACA8RD to the core of ACA8 (residues 131–1074, a construct lacking the regulatory domain) yields a dissociation constant (*K*_d_) of 1.7 μM (Fig. 2b). Addition of CaM to this preformed complex resulted in a decrease in anisotropy (*K*_0.5_ approx. 120 nM), indicating that the displacement of ACA8RD from the core of ACA8 is dependent on CaM-binding to ACA8RD (Fig. 2c). The displacement setup described in Fig. 2c reports indirectly on the binding of the second CaM molecule to the low-affinity binding site, as the regulatory domain will be only displaced from the ACA8core construct once both CaM are bound. Overall, these binding experiments indicate that the regulatory domain binds weakly to the core of the ACA8 pump. Binding of two CaM molecules to regions on the regulatory domain that are also involved in binding to the catalytic core^12^ can then displace the regulatory domain from the core enzyme. The fact that the regulatory domain has a higher affinity to CaM than to the core enzyme suggests that the availability of Ca^2+^-loaded CaM is the dominant factor in the regulation of pump activity.

Displacement of the regulatory domain caused by CaM-binding can also be observed by size-exclusion chromatography (SEC). The SEC-profile of the ACA8 + CaM complex in ND reveals a shift of the elution peak (11.1 ml for complex vs. 11.9 ml for ACA8 alone) that cannot be simply explained by the binding of CaM but rather indicates a hydrodynamic expansion of the complex in solution leading to this drastic change in the particle elution volume (Fig. 1d).

**Fig. 3:**
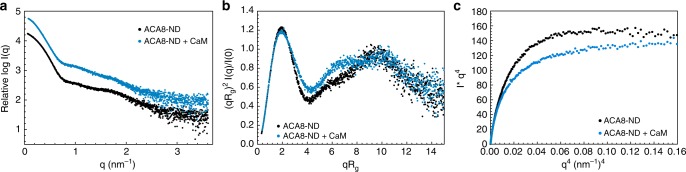
Small-angle X-ray scattering (SAXS) analysis of nanodisc-incorporated ACA8 and its complex with CaM. **a** Binding of CaM to ACA8 leads to an increase in *R*_g_ and *D*_max_. **b** Dimensionless Kratky plot of the data in **a** highlighting the conformational change of the regulatory domain upon CaM binding to ACA8. **c** Porod-Debye-Plot plot without plateau for the ACA8-CaM complex indicating a decrease in the overall contrast due to a more flexible conformation. Data for ACA8 in ND are depicted in black, data for the corresponding complex with CaM are shown in blue

With these data in hand the structural changes during activation were further studied by small-angle X-ray scattering (SAXS). SAXS is a suitable technique to determine overall dimensions and shapes as well as flexibility and larger conformational changes of biomolecules in solution. We performed SAXS measurements of POPC-nanodisc-incorporated ACA8 and its complex with CaM (Fig. 3). The calculated *M*_r_ from *I*(0) values are in excellent agreement with the theoretical values (Table 1). Binding of CaM leads to an increase in radius of gyration (*R*_g_) from 5.3 nm for the apo ACA8 to 5.88 nm for the CaM-activated state and a slight increase in maximum diameter (*D*_max_) (20 nm/22 nm) once CaM is bound (Table 1/Supplementary Fig. 4). Beside the increase in *R*_g_ and *D*_max_ the dimensionless Kratky plot ((qR_g_)^2^ x I(q)/I(0) vs. qR_g_)  shows differences in the mid q-range where usually domain movements are visible, indicating conformational rearrangement and increased flexibility, which is most likely due to the displacement of the regulatory domain upon CaM binding (Fig. 3b). This data is in line with the fluorescence anisotropy data and shows that the conformational changes upon activation of ACA8 by CaM can be detected by small-angle X-ray scattering. Moreover, we analyzed the flexibility of the regulatory domain in the activated state using the Porod-Debye plot. A plot of *q*^4^*I*(*q*) vs. *q*^4^ will achieve a plateau for more compact molecules and not for more flexible molecules. The ACA8-CaM complex does not show a plateau in the low q-range of the Porod-Debye plot (*q*^4^ < 0.1 nm^−4^), indicative of a more diffuse contrast that is consistent with a flexible conformation. A clear plateau is visible for ACA8 in its autoinhibited state (Fig. 3c). SAXS measurements with ACA8 incorporated in nanodiscs containing anionic lipids (POPG or soy-PI) did not show any expansion in the absence of CaM with radii comparable to those of ACA8 in POPC-nanodiscs (Supplementary Fig. 5). Binding of CaM to ACA8 in nanodiscs containing anionic lipids leads to an increase in *R*_g_. This indicates that anionic lipids are not sufficient to fully activate the pump on their own, however they seem to play a role in later stages of CaM activation. We speculate that anionic lipids might stabilize an intermediate conformation that can readily be activated by CaM-binding leading to high activity gain (Fig. 1a).

**Table 1:** SAXS parameters

	ACA8-ND (apo)	ACA8-ND + CaM
	SAXS	SAXS
	(SASDEV4)	(SASDEW4)
*Data collection parameters*		
Instrument	EMBL P12 (PETRA-III, DESY, Hamburg)	
Beam geometry	0.2 × 0.12 mm^2^	
Wavelength (Å)	1.24	
*q* range (nm^−1^)	0.05–4.6	
Exposure time (s)	1 (20 × 0.05 s)	
Concentration range (mg/ml)	1–8	1–6
Temperature (K)	283	283
*Structural parameters*		
*I*(0) (cm^−1^) [from *p(r)*]	0.093	0.129
*R*_g_ (Å) [from *p(r)*]	53.8	60.0
*I*(0) (cm^−1^) (from Guinier)	0.094	0.128
*R*_g_ (Å) (from Guinier)	53.0 ± 0.9	58.8 ± 1.9
*D*_max_ (Å)	200	220
Porod volume estimate (Å^3^)	626450	805590
*Molecular-mass determination*		
Partial specific volume (cm^3^ g^−1^)	0.816^a^	0.810^a^
Contrast (Δp × 10^10^ cm^−2^)	1.808	1.876
Molecular mass *M*_r_ (Da) [from I(0)]	259000	334000
Molecular mass *M*_r_ (Da) [from Porod volume (*V*_p_/1.6)]	391531	503493
Calculated *M*_r_ from sequence (Da)	256240^a^	288240^a^
*Software employed*		
Primary data reduction	RADAVER	
Data processing	PRIMUS/Qt	
Computation of model intensities	CRYSOL	
3D graphics representations	PyMOL	

^a^Calculated for a complex composed of 1xACA8 + 2xMSP1D1 + 124xPOPC molecules. This assumption is based on a homology model of ACA8 incorporated into MSP1D-POPC nanodiscs using the CHARMM GUI web server^45^

### Structural model of activated ACA8-(CaM)_2_ complex in nanodiscs

**Fig. 4:**
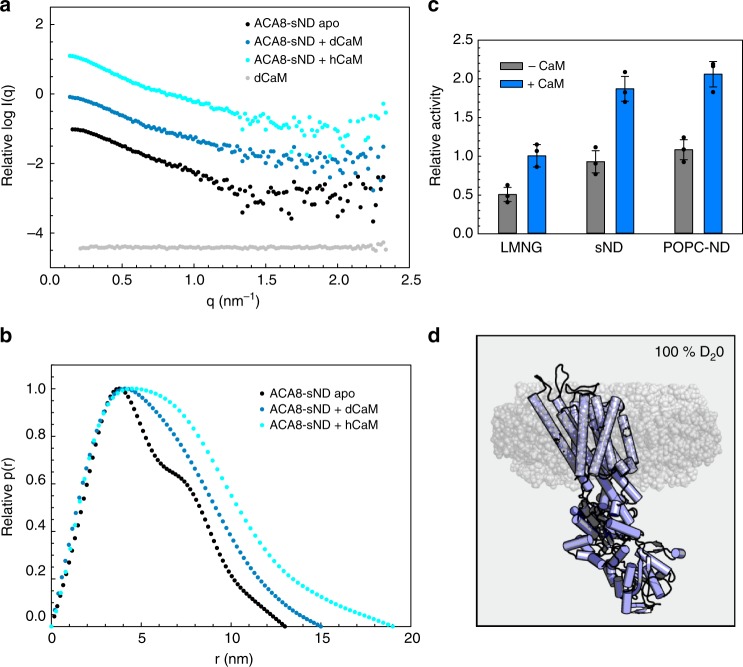
Small-angle neutron scattering (SANS) analysis of ACA8 and its ACA8-CaM complexes in stealth carrier nanodiscs. **a** Comparison of SANS scattering data of ACA8 in sND in 100% D_2_O in apo conformation (black) with data for the corresponding complex with hydrogenated (hCaM) and deuterated CaM (dCaM) (light blue and dark blue, respectively). The deuterated nanodisc components do not contribute to the SANS scattering signal in 100% D_2_O^18^. Scattering signal for deuterated CaM (73% deuterated) is shown in gray, showing that dCaM is fully matched out. **b** Distance distribution (P(r))-plot of the data shown in **a** indicating that binding of CaM to ACA8 leads to an increase of *D*_max_. **c** Activity assay in absence and presence of CaM. **d** Cartoon illustration of the used stealth carrier nanodisc technique. At 100% D_2_O the nanodisc components are fully matched out (shown in semi-transparent gray) and only ACA8 (violet) contributes to the scattering signal

In order to investigate the structure of ACA8 in its autoinhibited as well as CaM-activated state in a native-like lipid environment we made use of the recently developed stealth nanodisc (sND) technology (Fig. 4d)^17,18^. Stealth nanodiscs are fractionally deuterium labeled scaffolds consisting of phosphatidylcholine lipids and MSP1D1 belt protein; these can be rendered effectively invisible to low-resolution neutron diffraction using the appropriate solvent contrast and allow the low-resolution structure determination of integral membrane proteins in a lipid environment without contribution of the scaffold to the small-angle neutron scattering (SANS) signal (Fig. 4d). Using this stealth nanodisc / SANS method structural changes involving the incorporated membrane protein are usually easier to detect and to model compared to SAXS experiments, as in the sND/SANS setup only the components of interest contribute to the scattering signal, while in the SAXS setup the nanodisc also contributes to the signal. The SAXS measurements, however, provide important independent controls, as the overall structural trends (such as increase in *R*_g_, *D*_max_, flexibility etc.) should also be observable.

Control experiments confirmed that the deuterated disc constituents do not affect the activation process of ACA8 by CaM, as the expansion in hydrodynamic radius as well as the activity is comparable to those in non-deuterated nanodiscs (Fig. 4c; Table 2). SANS data were acquired (all in sND) (1) for apo-ACA8, (2) ACA8 in complex with protonated CaM, and (3) ACA8 in complex with perdeuterated CaM (dCaM), where only ACA8 in its activated state contributes to the scattering signal as the contrast matched-out CaM is invisible to neutron scattering (Fig. 4). From the SANS scattering data we could calculate parameters of overall size (*R*_g_, *D*_max_) that clearly show expansion of ACA8 upon CaM-binding (Table 2/Supplementary Fig. 6).

**Table 2:** SANS parameters

	ACA8-sND (apo)	ACA8-sND + dCaM	ACA8-sND + CaM
	SANS	SANS	SANS
	(SASDES4)	(SASDET4)	(SASDEU4)
*Data collection parameters*			
Instrument	SANS-1 (MLZ, Munich)	SANS-1 (MLZ, Munich)	SANS-1 (MLZ, Munich)
Wavelength (Å)	5.0	5.0	5.0
q range (nm^−1^)	0.12–2.4	0.12–2.4	0.12–2.4
Exposure time (s)	7200	7200	7200
Concentration (mg/ml)	3.5	3.2	3.0
Temperature (K)	283	283	283
*Structural parameters*			
*I*(0) (cm^−1^) [from *p(r)*]	0.11	0.1	0.15
*R*_g_ (Å) [from *p(r)*]	42.5	45.8	52.9
*I*(0) (cm^−1^) (from Guinier)	0.11	0.092	0.1
*R*_g_ (Å) (from Guinier)	40	42.8	50
*D*_max_ (Å)	130	150	180
Porod volume estimate (Å^3^)	202000	217000	297000
*Molecular-mass determination*			
Partial specific volume (cm^3^ g^−1^)	0.744	0.744	0.739
Contrast (Δp x 10^10^ cm^−2^)	−3.320	−3.320	−3.299
Molecular mass *M*_r_ (Da) [from Porod volume (*V*_p_/1.6)]	126250	135625	185625
Calculated *M*_r_ from sequence (Da)	118000	118000	150000
*Software employed*			
Primary data reduction	BerSANS	BerSANS	BerSANS
Data processing	PRIMUS/Qt	PRIMUS/Qt	PRIMUS/Qt
Rigid-body modeling		EOM	
Computation of model intensities	CRYSON	CRYSON	CRYSON
3D graphics representations	PyMOL	PyMOL	PyMOL

The experimentally derived radius of gyration for ACA8 in stealth nanodiscs was 4.0 nm for the apo conformation, and the distance distribution function has a bi-lobed shape with two peaks, at 3.6 and 7.0 nm, and a maximal particle dimension of 13 nm, which is consistent with a rather compact multi-domain particle (Fig. 4b). Once CaM binds to ACA8 both *R*_g_ and *D*_max_ show a large increase to 4.3 nm and 15 nm, respectively, for the complex with deuterated CaM, which is in line with the SAXS data. The complex of ACA8 with hydrogenated CaM shows an even bigger *R*_g_ and *D*_max_ of 5.0 nm and 18 nm, respectively, indicating that the deuterated CaM is effectively matched out in the ACA8-sND + dCaM complex (Fig. 4a, b; Table 2). The peak at 7.0 nm in the distance distribution function becomes less pronounced and shifts towards larger distances when either hydrogenated or deuterated CaM are used, indicative of protein expansion. These data are consistent with the model that the binding of the regulatory domain to the core is released and the ACA8 structure becomes less compact relative to the apo conformation (Fig. 4b), as already indicated by the SAXS experiments. Notably, all *R*_g_ values derived from SANS measurements in sND are smaller than those obtained by SAXS indicating the minimal scattering contribution of the nanodisc to the SANS data (Tables 1 and 2). For this reason, the observed changes upon activation by CaM are more pronounced in our SANS data using sND compared to the SAXS data.

**Fig. 5:**
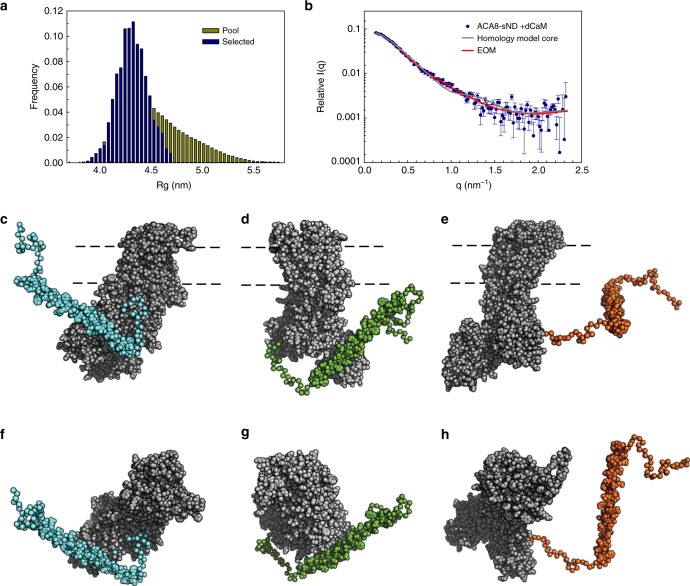
Structural model of the activated ACA8-(CaM)_2_ complex. The Ensemble Optimization Method (EOM)^20^ was used to generate and select a pool of models with different conformations of the regulatory domain (**a**) that were subsequently used to fit the experimental SANS data (acquired in stealth nanodiscs) (**b**). **c**–**h** The resulting representative models for the activated ACA8-(CaM)_2_ complex from the best-fitting sub-ensemble show ACA8core (gray) with the regulatory domain in three different conformations (cyan, green and orange). **f**–**h** are rotated by 90 degrees

In order to generate a structural model for ACA8 in its activated CaM-bound state, high-quality ACA8 + dCaM stealth nanodisc data were obtained from SANS measurements. With the activating CaM and lipid nanodiscs rendered invisible due to the partial deuteration strategy employed, the acquired dataset represents a fully-activated ACA8 in a native-like lipid environment. We generated a homology model of the “ACA8 core” (residues 130–1074) based on a sarco/endoplasmic reticulum Ca^2+^-ATPase (SERCA) structure in E2 conformation (pdb:3b9b). We used the long helix (residues 40–95) from the previously determined crystal structure of the ACA8 regulatory domain in complex with CaM^9^ (pdb:4aqr) and generated models of the linker (96–130) and the N-terminal extension (1–40) in various conformations using the program RANCH^19,20^. The quality of these models was evaluated by back-calculating the scattering intensities from the models and fitting against the measured SANS data of the complex with deuterated CaM (Fig. 5). Although the overall size and shape of the homology model complexes describe the low-q regions of the SANS data well, no single model was found to provide a satisfactory fit to the experimental data across the entire data range. As the regulatory domain is expected to be more flexible in the activated state we performed ensemble optimized modeling (EOM) analysis to quantitatively assess changes in the ensemble-averaged conformation upon CaM binding. The use of EOM for the modeling of SANS data acquired for integral membrane proteins in stealth nanodiscs is a very promising strategy for the structural characterization of flexible IMPs. EOM uses a genetic algorithm to select an ensemble of conformers of the regulatory domain whose weighted averaged scattering curve best reproduces the experimental scattering data^19,20^. The selected ensemble (best performing sub-ensemble) reveals an excellent fit to the experimental data (chi^2^ = 1.0) (Fig. 5b). Three representative models with *R*_g_ values between 4.0 and 4.4 nm show different orientations of the regulatory domain (Fig. 5c) and as an ensemble describe the conformational flexibility of the complex. None of the selected conformers contains a fully extended regulatory domain, which is also apparent in the *R*_g_ distribution plot that is shifted to smaller *R*_g_ values for the selected pool compared to the random pool. The *R*_g_ size-distribution indicates that the complex occupies a restricted range of conformations that tend towards the more compact rather than extended conformations, relative to the random pool. This is also reflected in the metric *R*_flex_ that provides a quantitative measure for the flexibility of the system, with *R*_flex_ being 68.1% for the selected ensemble and 86.1% for the pool.

## Conclusions

Taken together, these data illustrate that CaM-binding to ACA8 leads to displacement of the regulatory domain from the ACA8 core enzyme. This displacement of the regulatory domain releases the autoinhibition and allows the enzyme to undergo its conformational cycle required for the pumping of Ca^2+^ ions. In combination with our previous crystal structure of the ACA8 regulatory domain in complex with CaM^9^, this structural model for activated ACA8 reveals the structural basis for activation of plasma-membrane Ca^2+^-ATPase by calmodulin in the context of the full-length protein in a native-like lipid environment. The results add further structural insights into the previously proposed bimodular activation mechanism^9^ showing that binding of two CaM molecules leads to full activation of this Ca^2+^ pump via displacement of the regulatory domain.

## Methods

### Materials

Detergents and lipids were purchased from the following companies: DDM (#D310/Anatrace), LMNG (#NG310/Anatrace), cholate (#3407.1/Roth), POPC (#850457 P/Avanti Polar Lipids), POPG (#840457P/Avanti Polar Lipids), soyPI (#840044P/Avanti Polar Lipids). All other chemicals were of analytical grade and obtained from Roth (Karlsruhe, Germany) or Sigma Aldrich (St. Louis, MO, USA).

### Expression and purification of full-length ACA8, ACA8core, and ACA8RD

ACA8 was overexpressed with an N-terminal His_8_-tag in *S. cerevisiae* strain BJ5460 (MATa ura352 trp1 lys2801 leu2delta1 his3delta200 pep4::HIS3 prb1delta1.6R can1 GAL)^21^ using a pYES2 plasmid. Large-scale cultures were grown in uracil-depleted media (6.7 g l^−1^ YNB) + 0.1% glucose at 30 °C up to an OD_600_ of 0.6 and expression was induced by adding 2% galactose. Cells were harvested 20 h post induction by centrifugation at 3000x*g* and resuspended in 30 mM Tris pH 8.0, 300 mM NaCl, 20% (v/v) glycerol, 3 mM ß-mercaptoethanol, 20 mM EDTA (buffer A) before being disrupted with glass beads. Following clearing of the lysate, cell membranes were isolated by centrifugation at 180,000×*g* and membranes were solubilized in buffer A with 1% lauryl maltose neopentyl glycol (LMNG) for 1.5 h with gentle stirring. To remove insoluble material the solubilized membranes were centrifuged at 100,000×*g* and supernatant were incubated with Ni^2+^ affinity resin. Resin was washed with buffer containing 30 mM Tris pH 8.0, 300 mM NaCl, 2 mM CaCl_2_, 1 mM ß-mercaptoethanol, 0.005% LMNG and 40 mM imidazole and ACA8 were eluted with 150 mM imidazole. The purity of ACA8 was judged by SDS-PAGE and corresponding fractions were pooled and concentrated to 2 mg/ml before reconstituted into nanodiscs. The ACA8 mutant lacking the regulatory domain (ACA8core) was expressed and purified in the same way as the full-length protein.

The regulatory domain of ACA8 (aa 1–130) (ACA8RD) was cloned as a fusion construct with an N-terminal His_6_-lipoamyl-TEV-tag^22^ in the pET28a vector and a cysteine was introduced right before the first residue of the regulatory domain to allow site specific labeling with thiol-reactive probes. ACA8RD was co-expressed together with CaM7 in *E. coli* strain C41^23^, grown in 2xTY media at 20 °C for 16 h. Cells were harvested at 4000×*g* and resuspended in buffer containing 30 mM Tris pH 7.5, 300 mM NaCl, 10% glycerol, 2 mM CaCl_2_, 1 mM Tris-(2-carboxyethyl)-phosphin (TCEP) (buffer D) before lysed using high-pressure homogenizer (EmulsiFlex-C3, Avestin). The cleared lysate was loaded on HisTrap column, pre-equilibrated with buffer D and washed with 20 mM imidazole before eluted with 200 mM imidazole followed by TEV protease digestion and a second nickel affinity chromatography step to separate the His-lipoamyl-TEV tag.

### Expression and purification of CaM7

CaM7 from *A. thaliana* was used as calmodulin ortholog throughout this study. CaM7 in pET42a vector was transformed into *E.coli* Bl21 Gold (DE3) and grown in LB medium at 37 °C. After induction of protein expression with 0.5 mM isopopropyl ß-D-1-thiogalactopyranoside (IPTG), cells were grown for another 16 h at 20 °C and harvested by centrifugation at 3000 × *g*. Cells were resuspended in 30 mM Tris pH 7.5, 50 mM NaCl, 1 mM ß-ME, 2 mM CaCl_2_ (buffer B) and broken using high-pressure homogenizer (EmulsiFlex-C3, Avestin). The lysate was cleared by centrifugation at 40,000 × *g* and the supernatant was bound to HiTrap Phenyl HP column, pre-equilibrated with buffer B. CaM7 was eluted with 5 mM EDTA. Fractions containing pure CaM7 were pooled, concentrated to 10 mg/ml and stored at −80 °C until further use.

### Expression and purification of membrane scaffold protein MSP1D1

MSP1D1 in pET28a vector was transformed in *E.coli* strain BL21 (DE3) and grown in *terrific broth* (TB) media at 37 °C. At an OD_600_ of 1.5 the protein expression was induced by adding 1 mM isopropyl ß-d-1-thiogalactopyranoside (IPTG) and cells were grown for 4 h at 37 °C. The protein was purified according to a modified protocol established by Sligar and co-workers^24^. Briefly, cells were harvested by centrifugation at 3000×*g*, resuspended in lysis buffer (50 mM Tris pH 8.0, 500 mM NaCl) with 1% Triton X–100 and broken using sonication. The cleared lysate was loaded onto a HisTrap column and washed with ten column volumes each of lysis buffer containing 1% Triton X-100 and 50 mM cholate, respectively. MSP1D1 was eluted with buffer containing 500 mM imidazole, and fractions containing pure protein were pooled and incubated with TEV protease overnight. Subsequently, the protease and cleaved His-tag were separated by applying a second IMAC chromatography step and MSP1D1 without His-tag was concentrated up to 400 µM and stored at −80 °C until further use.

### Expression and purification of deuterated membrane scaffold protein MSP1D1 (dMSP1D1), deuterated phosphatidylcholine (dPC), and deuterated calmodulin (d-CaM7)

Matchout-labeled MSP1D1 (d-MSP1D1) was overexpressed in *E. coli* strain BL21 (DE3) as previously described^25,17^. After adaption of the strain to minimal deuterated medium^26^, cultures were grown in flaks in 85% deuterated minimal medium with glycerol as a carbon source^27^. The protein was purified according to a modified protocol established by Sligar and co-worker^24^ (as described above for non-deuterated protein).

Selectively deuterated mixed acyl phosphatidylcholine, PC, was produced in a modified *E. coli* strain grown in minimal 100% deuterated medium supplemented with deuterated glycerol (C3D8O3) and partially deuterated choline chloride (trimethyl- d9, 98%; Eurisotop) as previously described^28^. Total phospholipids were extracted using the method of Bligh and Dyer^29^ and purified according to head-group using silica-gel column chromatography with varying ratios of chloroform and methanol as previously described^28^.

Matchout-labeled CaM7 (d-CaM7) with a deuteration level of 73% was overexpressed in *E. coli* strain BL21 (DE3) adapted to growth in deuterated minimal medium^30^. A 1.8 l (final volume) deuterated high cell-density fed-batch fermenter culture^25^ was carried out at 30 ^ο^C. Feeding with glycerol was started at an OD_600_ value of about 5. Expression of d-CaM was induced at an OD_600_ of about 12 by addition of IPTG (0.5 mM final concentration). Cells were harvested at an OD_600_ of 19 yielding 100 g wet weight of matchout-labeled cell paste. Cells were resuspended into buffer containing 30 mM Tris pH 8.0, 100 mM NaCl, 1 mM CaCl_2_, lysed using a high-pressure homogenizer (EmulsiFlex-C3) and purified by hydrophobic affinity chromatography (using a HiTrap Phenyl HP column), as described above.

### Reconstitution of ACA8 into (stealth) nanodiscs

For reconstitution of ACA8 or ACA8core into nanodiscs, 50 mM lipids (1-palmitoyl-2-oleoyl-*sn*-glycero-3-phosphocholine (POPC), 1-palmitoyl-2-oleoyl-sn-glycero-3-phospho-(1’-rac-glycerol) (POPG) or L-α-phosphatidylinositol (soy-PI) (Avanti Polar Lipids)) were dissolved in a buffer containing 30 mM Tris pH 8.0, 200 mM NaCl, 2 mM CaCl_2_, 1 mM ß-mercaptoethanol (buffer C) and 100 mM cholate. Lipid mixtures contained 70% POPC and 30% POPG or 70% POPC and 30% soy-PI, respectively. ACA8, the MSP1D1 membrane scaffold protein and the lipids were mixed in a molar ratio of 1:5:150 in buffer C containing 20 mM cholate and incubated for 1 h at 4 °C. By adding detergent removal beads (Thermo Fischer Scientific) in a 1:1 (v/v) ratio, detergents were removed to initiate the nanodisc assembly and the sample was incubated over night at 4 °C under constant agitation. Detergent removal beads were removed and the sample cleared by centrifugation before subsequent purification of nanodisc-embedded ACA8 on a Superdex200 column (GE Healthcare) in a buffer containing 20 mM Tris pH 8.0, 150 mM NaCl, 1 mM CaCl_2_, 1 mM ß-mercaptoethanol. For reconstitution of ACA8 in stealth carrier nanodiscs (sND) the molar ratio of all components and the protocol was kept unchanged, but deuterated MSP1D1 and deuterated PC were used and the assembled stealth nanodiscs were dialyzed extensively against D_2_O-based buffer.

### Native mass spectrometry (NMS)

Detergent (DDM)-solubilized ACA8 was exchanged to 200 mM ammonium acetate solution pH 8.3, 2x CMC (0.018%) DDM using centrifugal filter units (Vivaspin 500, 100,000 MWCO, Sartorius) at 4 °C and 15,000×*g*. Nano electrospray ionization (ESI) capillaries were prepared as described before^31^.

Native MS experiments were performed with a nanoESI source in positive ion mode on a QToF2 (Waters and MS Vision) that was modified for the analysis of high-mass ions^32^. 7 mbar source pressure and 1.7 × 10^–2^ mbar argon as collision gas were used. Capillary and cone voltages were set to 1.7 kV and 190 V, respectively. The collision energy was ramped up to 400 V, the illustrated spectra were recorded at 200 V. CsI (25 mg/ml) spectra were acquired and used to calibrate raw data using MassLynx software (Waters). Data were analyzed using MassLynx and Massign^33^.

### Activity measurements

ACA8 activity was measured either in LMNG or nanodisc composed of different lipids as well as stealth carrier nanodisc using the Baginski assay^34^. All reactions were performed in buffer containing 150 mM NaCl, 30 mM Tris-HCl (pH 7.4 at 25 °C), 2 mM MgCl_2_, 1.95 mM EGTA, and 2 mM CaCl_2_, resulting in 50 μM final free Ca^2+^ concentration. Three microgram of purified ACA8 in LMNG and 2 µg ACA8 in nanodisc were incubated in 50 µl sample buffer with 1 mM ATP for 10 min at 25 °C before the reaction was stopped by adding 50 µl ascorbic acid solution (140 mM ascorbic acid, 0.5 M HCl, 0.1% SDS, 5 mM ammonium heptamolybdate). The addition of 75 µl containing 75 mM sodium citrate, 2% (w/v) sodium metaarsenite and 2% (v/v) acetic acid stopped the colorimetric reaction and the absorbance at 860 nm was read on a Tecan Infinite200 microplate reader after 10 min. Calmodulin was added in a range between 5 nM and 10 µM prior ATP addition. All activity measurements were performed under initial velocity conditions. The spontaneous and non-enzymatic hydrolysis of ATP just in buffer was subtracted from the measurements with ACA8. Mixed lipids contained 70% POPC and 30% POPG or 70% POPC and 30% soy-PI, respectively. A calibration curve using sodium phosphate in a concentration range from 0.01 to 0.6 mM was used for determining the concentration of released phosphate. All reactions were measured as triplicates.

### Fluorescence anisotropy titration

For fluorescence anisotropy measurements Alexa Fluor 488 C_5_ maleimide was used to label ACA8RD or CaM. For fluorescence labeling Alexa Fluor 488 C_5_ maleimide were added to 40 µM ACA8RD-(CaM7)_2_ or 40 µM CaM, respectively, in a molar ratio of 10:1 and incubated overnight at 4 °C. The reaction were stopped by adding 5 mM ß-mercaptoethanol, free fluorescein isothiocyanate (FITC) was separated with a PD10 column and the ACA8RD-(CaM)_2_ complex were dissociated by adding 10 mM EDTA followed by binding of ACA8RD to a cation exchange chromatography column to separate both proteins. Fractions containing ACA8RD were pooled, concentrated and flash frozen in liquid nitrogen until further use. FITC-labeled CaM was used directly after PD10 column. Measurements were performed on an Agilent Cary Eclipse fluorescence spectrophotometer. Fluorescence anisotropy was measured with excitation at 480 nm and emission at 520 nm and slit width of 10 nm. Each measurement was integrated over 5 s and the photomultiplier voltage was set to 700 V. Reactions were carried out at 20 °C in buffer containing 30 mM Tris pH 8.0, 150 mM NaCl, 2 mM CaCl_2_, 0.5 mM TCEP. In the binding experiments, 25 µM ACA8core (in POPC nanodiscs) were titrated into 50 nM fluorescein-labeled ACA8RD or full-length ACA8 (in POPC nanodiscs) was titrated into 15 nM FITC-labeled CaM. Dissociation constants were obtained by fitting anisotropy data to the equation corresponding to a one-site binding model with *r*_obs_ = *r*_0_ + (Δr × [P]) / (*K*_d_ + [P)]

### Small-angle X-ray scattering (SAXS)^18^

SAXS was measured using the Bio-SAXS instrument P12 on the storage ring Petra III (DESY, Hamburg, Germany)^35^. The scattered intensity was recorded as a function of the scattering vector *q* with $\left| q \right|$ = 4 $\pi$ sin*θ*/λ, using a wavelength of 0.124 nm. All SAXS measurements were carried out at 10 °C in 30 mM Tris pH 7.5, 150 mM NaCl, 2 mM CaCl_2_, 2 mM MgCl_2_ and 0.5 mM TCEP at protein concentrations ranging from 1 to 8 mg/ml with exposure times of 20 × 0.05 s. The average of the data was normalized and background subtracted using automatic procedures on the beamline^36^. Calibration of the scattering intensity into absolute units of cm^−1^ was performed using the forward scattering intensity of bovine serum albumin. The radius of gyration was evaluated from the experimental SAXS pattern using the Guinier approximation and as well as from the entire scattering curve using the program GNOM^37^. The latter also provided the distance distribution function, *p*(*r*), and the maximal dimension, *D*_max_ (see Table 1)_._

### Small-angle neutron scattering (SANS)^18^

SANS data of ACA8 in the autoinhibited as well as in the activated state (with hydrogenated and deuterated calmodulin) were collected at the SANS-1 beamline at Forschungs-Neutronenquelle Heinz Maier-Leibnitz (FRMII) in Munich^38^. All measurements were performed in 100% D_2_O buffer (30 mM Tris pH 7.5, 150 mM NaCl, 1 mM MgCl_2_, 1 mM CaCl_2_) using a sample concentration of 3.0–3.5 mg/ml at 10 °C. Measurements at FRMII were performed at 5 Å wavelength with a sample-detector distance of 5.5 m (0.01 < *q* < 0.23 Å^−1^) where the detector was moved by 404 mm in direction perpendicular to the beam. Water reference sample (H_2_O), buffers, empty cell, the direct beam and the total absorber boron-cadmium were measured as well to perform data reduction using the BerSANS software and yielded one-dimensional scattering intensities *I*(*q*). The scattering curves of all samples were buffer subtracted using the software PRIMUS^36^ and the radii of gyration were extracted by the Guinier approximation. For SANS data sets at 100% D_2_O molecular mass estimates were obtained from the forward scattering (*I*_0_), with the contrast and partial specific volume as determined from the solution components and protein sequence using the MULCH server (http://smb-research.smb.usyd.edu.au/NCVWeb/)^39^. All SANS and SAXS scattering parameters have been included in Tables 1 and 2 according to the recommended publication guidelines for small-angle scattering studies^40^.

### Model calculation from SANS data (ACA8 in stealth carrier nanodiscs)

The conformational flexibility of ACA8 in its activated state was probed using the ensemble optimized modeling (EOM) program^20^. A homology model of ACA8core (130aa-1074aa) was generated with the program Phyre2^41^ using the SERCA structure in E2 conformation (pdb:3b9b). A pool of 10,000 ACA8 full-length models with the different conformations of the regulatory domain was generated with RANCH^20^ by using the ACA8core homology model and the crystal structure of the regulatory domain (pdb:4aqr) as input domains. Theoretical scattering intensity of each model was computed with CRYSON^42^. The genetic algorithm method, GAJOE^20^, from the EOM package was subsequently used to select a subset of models, whose weighted average scattering curve showed the best fit to the data of ACA8-dCaM complex (Fig. 5a / Supplementary Fig. 7). Structural models were displayed using PyMOL^43^.

## Electronic supplementary material

Supplementary Information

## Data Availability

The SAXS and SANS data have been deposited at the SASBDB (www.sasbdb.org) and have been assigned the following accession codes: SASDES4, SASDET4, SASDEU4, SASDEV4, SASDEW4 (see Tables 1, 2). The mass spectrometry data have been deposited to the ProteomeXchange Consortium via the PRIDE^44^ partner repository with the dataset identifier PXD011177. All other relevant data generated and/or analyzed during the current study are available from the corresponding author on reasonable request.
