# Supplementary material for: Structural basis for activation of plasma-membrane Ca2+-ATPase by calmodulin
Source: Commun Biol. 2018 Nov 26;1:206. doi: 10.1038/s42003-018-0203-7 (PMC6255812; doi:10.1038/s42003-018-0203-7)
Supplement: Supplementary file 1 — Supplementary Information [file 42003_2018_203_MOESM1_ESM.pdf]

Supplementary Figure 1

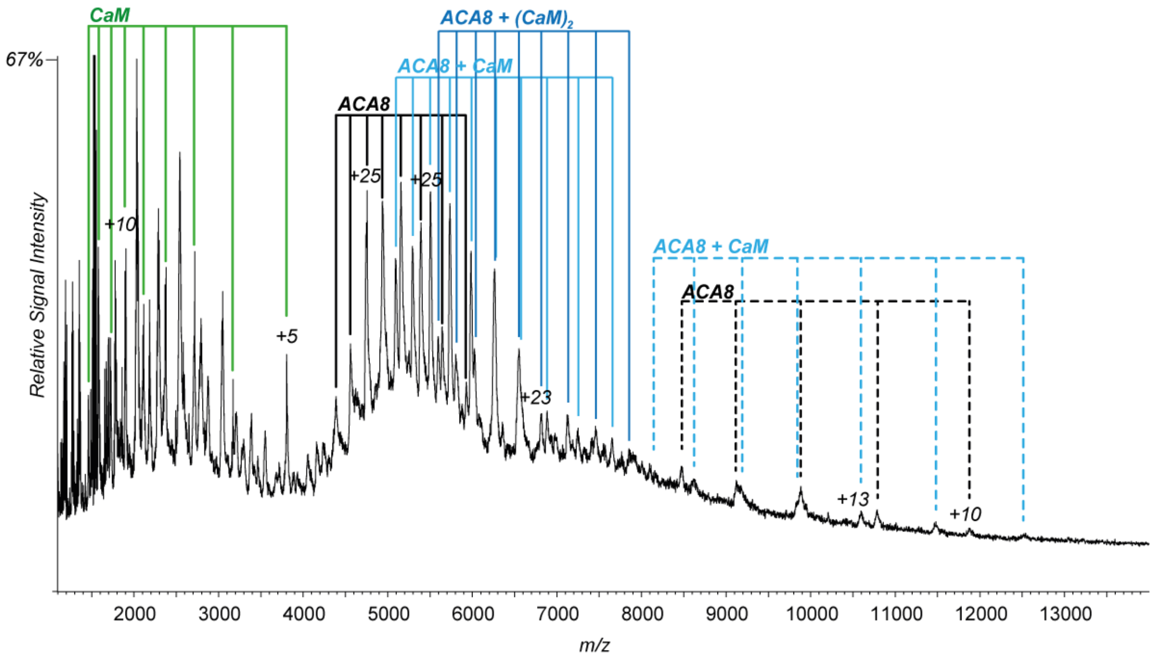

Full *m/z* range native mass spectrum shows up to two CaM molecules bound to full-length ACA8. With increased acceleration voltages ACA8 (solid black), ACA8+CaM (solid light blue) and ACA8+$(CaM)_2$ (dark blue) were released from DDM micelles. As a consequence of the collisions CaM can also dissociate from the CaM-bound ACA8, leading to highly charged CaM (green) and residual ACA8 (dotted black) and ACA8+CaM (dotted light blue).

Supplementary Figure 2

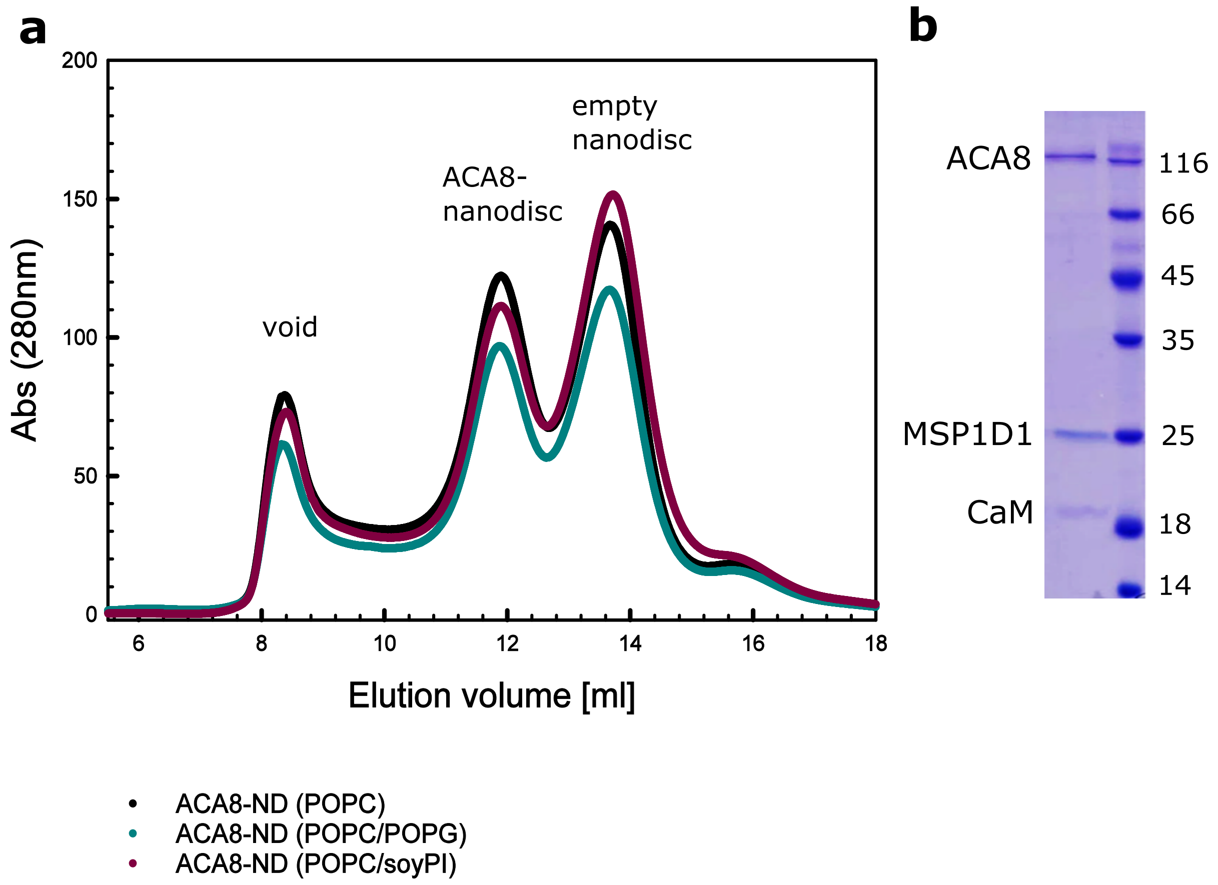

Sample purity of nanodisc incorporated ACA8 (and its complex with CaM)

A) Size-exclusion profile of purified ACA8 in nanodiscs formed with MSP1D1 and different lipids (POPC, POPC/POPG, POPC/soyPI). B) Coomassie-stained SDS-PAGE shows the peak fraction of ACA8 (118 kDa) incorporated into MSP1D1 (23 kDa) nanodiscs in complex with CaM (16 kDa).

Supplementary Figure 3

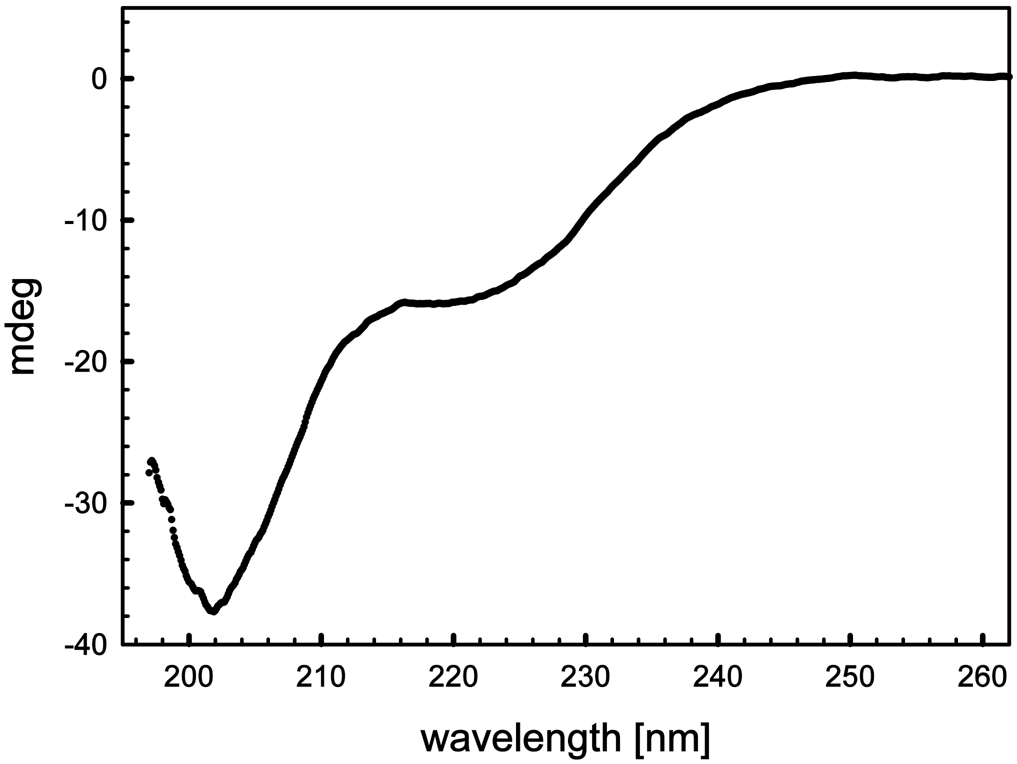

Far-UV CD spectrum of ACA8 regulatory domain (residues 1-130). Data were acquired using 15 µM ACA8RD in a buffer containing 50 mM sodium phosphate, pH 7.2 , 70 mM NaCl.

Supplementary Figure 4

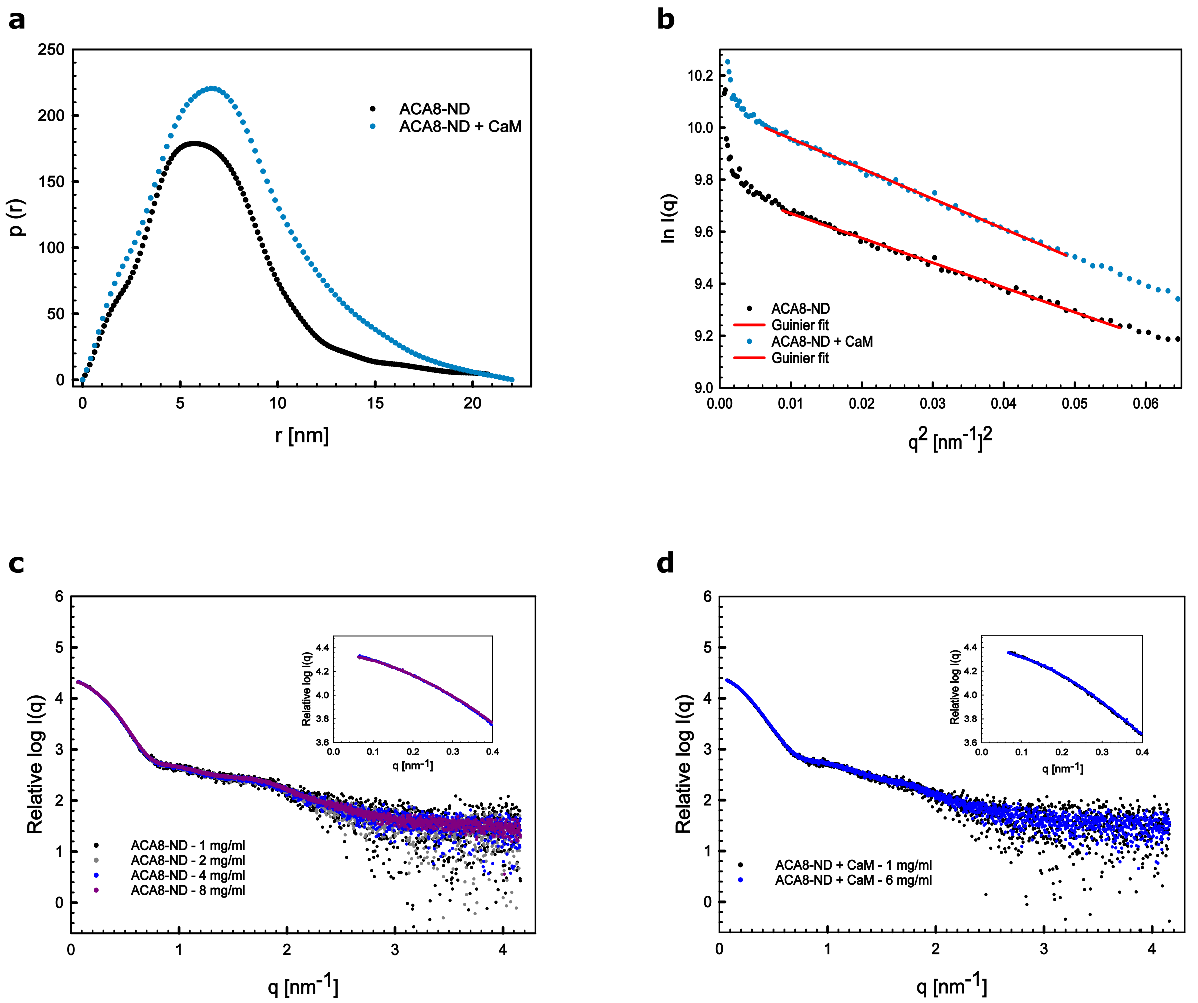

Small-angle X-ray scattering (SAXS) analysis of ACA8 incorporated in nanodiscs. A) Distance distribution plot of ACA8 in MSP1D1 nanodiscs in apo conformation (black) and in complex with CaM (blue) indicating that D_{max} increases once CaM is bound. B) Guinier region of the data shown in A) (apo conformation shown in black and in complex with CaM in blue) shows that the radius of gyration is increased when CaM is bound. First data points were affected by parasitic scattering and removed for data analysis. C-D) Overlay of SAXS profiles obtained for ACA8-ND (C) and ACA8-ND+CaM (D) in different concentrations. The curves superimpose very well; there is no indication for concentration dependence.

Supplementary Figure 5

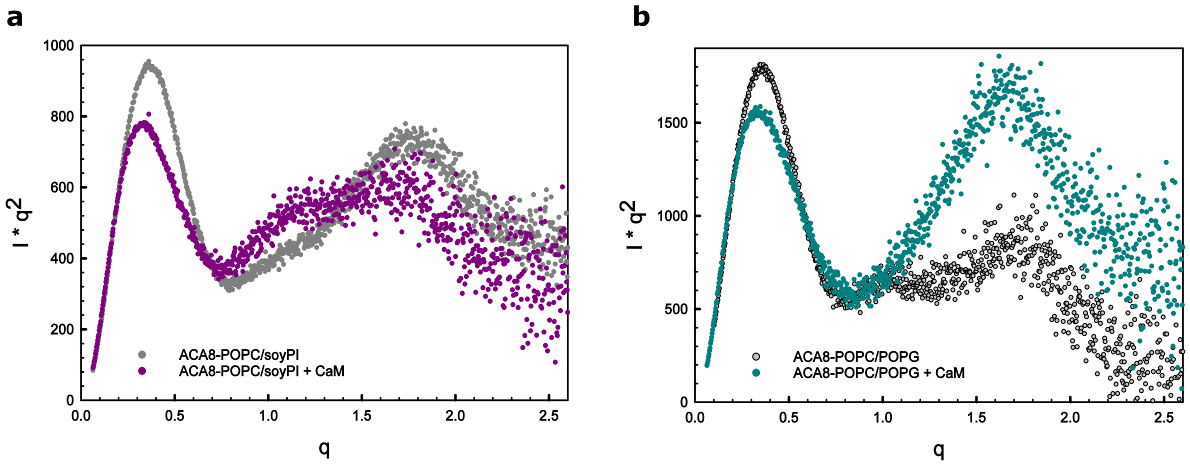

c

Radius of Gyration (Rg)	- CaM	+ CaM
ACA8-POPC	53.8 Å	60.0 Å
ACA8-POPC/soyPI	52.8 Å	60.0 Å
ACA8-POPC/POPG	53.0 Å	59.2 Å

Small-angle X-ray scattering (SAXS) analysis of ACA8 incorporated in nanodiscs containing anionic lipids. Kratky plots of ACA8 incorporated in nanodiscs containing soy-PI (A) or POPG (B) highlighting the conformational change of the regulatory domain upon CaM-binding to ACA8. C) Comparison of radii of gyration for various ACA8-nanodisc samples illustrating that the presence of anionic lipids does not lead to expansion in the absence of CaM with radii comparable to those of ACA8 in POPC-nanodiscs.

Supplementary Figure 6

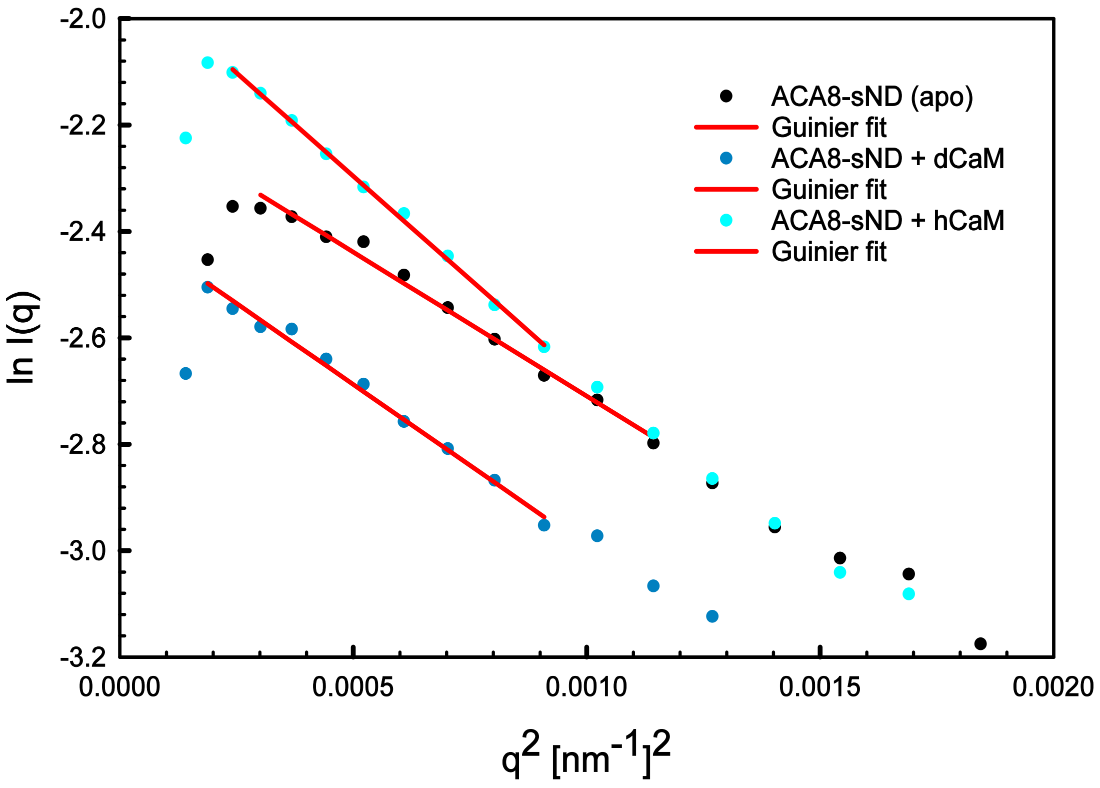

Guinier plots of SANS data showing the entire q-range. Guinier region of the SANS data obtained for ACA8 incorporated in stealth nanodiscs in apo form and in complex with deuterated and hydrogenated CaM. First data points were affected by parasitic scattering and removed for data analysis.

Supplementary Figure 7

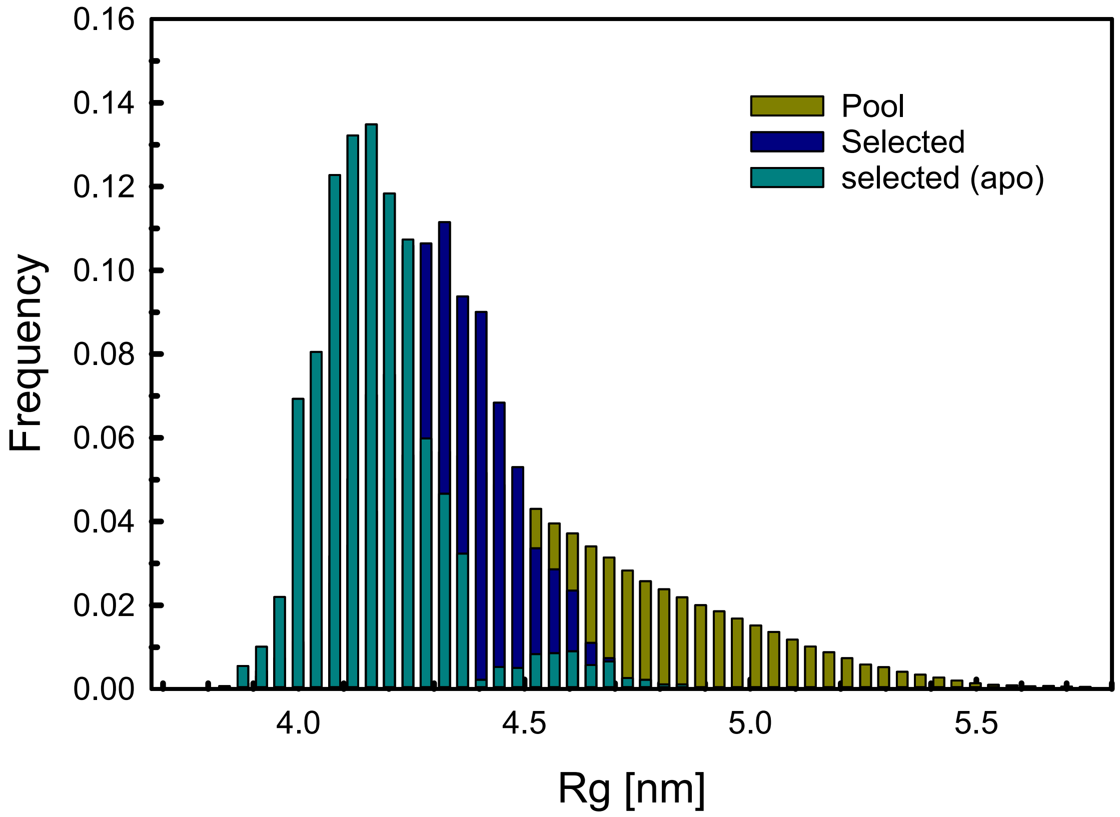

Ensemble Optimization Method (EOM) analysis. EOM analysis illustrating the R_g-distribution plot of ACA8 in apo conformation in comparison with the data of ACA8 in complex with CaM, showing that the selected sub-ensemble is significantly smaller and thus the R_g-distribution is shifted to smaller radii for ACA8 in the autoinhibited state.

Supplementary Table 1

Protein/-complex	Avg. Mass (Da)	St. dev. (Da, n≥3)	Avg. FWHM
**ACA8**	118520	60	804
**CaM**	18869.9	0.8	11
**ACA8 + CaM**	137500	110	700
**ACA8 + 2 CaM**	156600	200	730

Masses of ACA8 and ACA8-CaM complexes as determined by native MS. Averages from at least three measurements, standard deviation and FWHM (full width half maximum) of the main non-overlapping peaks are given.
